# Performance of DeepSeek and ChatGPT on the Chinese Health Professional and Technical Examination: A comparative study

**DOI:** 10.1371/journal.pone.0338328

**Published:** 2026-01-22

**Authors:** Xu Li, Xu Hu, Huiting Xu, Zhiang Sun, Pin Yu, Hailing Ju

**Affiliations:** 1 Department of Central ICU, The First Affiliated Hospital of Soochow University, Suzhou, China; 2 School of Medicine, Tongji University, Shanghai, China; 3 Department of Operating room, Qingpu Branch, Zhongshan Hospital Affiliated to Fudan University, Shanghai, China; 4 Department of Nursing, Tenth People’s Hospital Affiliated to Tongji University, Shanghai, China; South China Normal University, CHINA

## Abstract

**Background:**

Large language models (LLMs) are increasingly applied in medical education, yet their reliability in specialized, high-stakes assessments such as the Chinese Health Professional and Technical Examination remains unclear. DeepSeek-R1, a recently released reasoning-enhanced LLM, has shown promising performance, but empirical evidence within nursing examination contexts is limited.

**Objective:**

To compare the performance of DeepSeek-R1 and the GPT-4o API on the Chinese Health Professional and Technical Examination (Intermediate Nursing), focusing on accuracy, response consistency, and consistent accuracy.

**Methods:**

Four hundred official practice examination multiple-choice questions were categorized into four competency units and two question types (A/B). Both models were evaluated using overall accuracy, consistency (agreement across repeated responses), and consistent accuracy (proportion of responses that were both consistent and correct). Stratified analyses were performed across units, question types, and disciplines. Chi-square tests were used for statistical comparison, and Holm–Bonferroni correction was applied for multiple comparisons.

**Results:**

DeepSeek-R1 demonstrated significantly higher overall accuracy than the GPT-4o API (88.5% vs. 67.9%, P < 0.001). GPT-4o API showed higher response consistency (96.5% vs. 88.5%) but lower consistent accuracy (66.7% vs. 84.0%). After multiple-comparison correction, significant differences in consistent accuracy remained in basic knowledge, professional knowledge, professional practice ability and Type A questions, as well as in surgical and gynecological nursing disciplines, while other domains showed no statistically significant differences.

**Conclusion:**

DeepSeek-R1 outperformed the GPT-4o API across multiple dimensions of nursing competency assessment, particularly in overall accuracy and consistent accuracy. GPT-4o API exhibited high response stability but a tendency toward systematic errors, underscoring the need for careful interpretation of model outputs. Further research is needed to evaluate LLM performance using open-ended clinical reasoning tasks and real-world assessment data to support safe and effective educational integration.

## Introduction

With the rapid development of artificial intelligence technology, the application of large language models (LLMs) in nursing education, represented by ChatGPT, has gradually become a research hotspot. For example, Quattrini V et al. [1] used ChatGPT to generate teaching cases to evaluate the effectiveness of ChatGPT in nursing education, and the study found that ChatGPT was effective in improving students’ clinical judgment in nursing courses compared to traditional teaching methods. Musallam, E et al. [2] used ChatGPT for novel teaching of nursing simulation. The study found that nursing students can simulate patient communication and access relevant clinical information through the ChatGPT integrated system. Woodley, L. K. [3] and Simms, R. C. [4] incorporating ChatGPT into specific practice-based instruction, findings indicate that ChatGPT improves teaching effectiveness, promotes clinical skill development, and may also improve academic integrity, information validation, and clinical judgment in nursing students. While Vaughn J et al. [5] used ChatGPT to create accurate and realistic simulation scenarios for faculty assistants, the results of the study indicated that ChatGPT has the potential to help nursing educators develop clinical simulation scenarios to improve teaching and learning. At present, there are also many scholars applying LLMs such as ChatGPT to nursing knowledge test [6–10], The results of the studies all showed that LLMs were able to pass the examination criteria. LLMs became a very efficient tool for medical education [11].

As LLMs continue to be optimized, more and more researchers’ studies are not limited to mere knowledge quizzes, but rather to performance comparisons between different models. For example, Ming, S.’s [12] study found that the GPT-4o outperformed the GPT-3.5 in the key areas of accuracy, consistency, and medical subspecialty expertise on the Chinese Medical Licensing Examination. The performance of ChatGPT-3.5, ChatGPT-4 Omni (4o), Google Bard, and Microsoft Copilot in answering text-based multiple-choice questions (MCQs) related to oral radiology was evaluated and compared in a study by Tassoker, M. [13], and the results demonstrated that GPT-4o API has superior accuracy and advanced reasoning capabilities. Nowadays, most of the research is centered around ChatGPT, and there are fewer studies about DeepSeek’s application in medical exams or reasoning.

The DeepSeek-R1 model was officially launched by the Chinese company DeepSeek on January 20, 2025, and simultaneously open-sourced, causing a global sensation due to its problem-solving capabilities similar to those of ChatGPT [14]. Initially there have been studies conducted by scholars on the effectiveness of DeepSeek when used to communicate European Resuscitation Council (ERC) guidelines to the public with a non-medical background [15]. In a study by Birger Moëll et al. [16], DeepSeek R1 was demonstrated to have high clinical reasoning power through qualitative and quantitative analysis of 100 different clinical cases from the MedQA dataset. Whereas ordjman M et al. [17] who evaluated the performance of three models, LLM-DeepSeek-R1, ChatGPT-o1, and Llama 3.1-405B, on tasks in four different healthcare domains, showed that DeepSeek provided more accurate diagnostic reasoning steps than those provided by ChatGPT and Llama 3.1-405B. Waqas, A. et al. [18] evaluated the performance of four LLMs (OpenAI o1, OpenAI o3-mini, Gemini 2.0 Flash Thinking Experimental, and DeepSeek-R1 671B) on fifteen open-ended pathology problems, and found that DeepSeek was significantly better than OpenAI o1 in terms of overall reasoning quality significantly better than OpenAI o1 and OpenAI o3-mini, especially in terms of depth and consistency of analysis. However, a comparative study of the performance of DeepSeek and ChatGPT specifically on the Chinese Nursing Exam is not yet available.

The Chinese Health Professional and Technical Examination (Intermediate Level of Nursing) is the core standard for evaluating the professional competence and title promotion of Chinese nursing personnel, and the examination is a nationally standardized test that covers basic, internal medicine, surgical, obstetrics and gynecology, and pediatric nursing, as well as nursing management test questions. Existing studies have found that the accuracy of LLMs in basic knowledge questions in the Chinese nursing qualification exam can reach more than 90%, but the performance in complex application questions declines significantly, with an accuracy rate of less than 50% [19], while the design of questions in the Chinese Health Professional and Technical Examination (Intermediate Nursing) is characterized by higher specialization, stronger logic, and simulation of more complex clinical scenarios, and in the face of this discrepancy, the requirement of the accuracy of the model needs to match the depth of the exam design in order to be used as an effective teaching aid [20]. If LLMs have a high error rate in exams or generate incorrect answers in exams and are used for teaching, their clinical translation may lead to medical malpractice and possibly academic disputes [21]. Also, it has been shown that models with high accuracy reduce the risk of sensitive information leakage, while low-accuracy models may increase the probability of privacy exposure due to data noise [22] Existing studies have also noted that although the same test questions fed to LLMs in different languages can produce different results, the more advanced the model, the smaller the difference in different languages. ChatGPT 4.0 is able to handle both Chinese and English inputs well, and ChatGPT 4.0 consistently outperforms ChatGPT 3.5 and Google Bard in terms of accuracy as well [23] Gilson, A. et al. [24] replied in their study that although grammatical differences in English do lead to variability, there is no empirical evidence to suggest that this changes the performance of ChatGPT. This also tells us that applying LLMs in nursing exams we need to pay more special attention to the accuracy of LLMs.

Therefore, this study takes the China Health Professional and Technical Examination (Intermediate Nursing) exam simulation question bank as a benchmark, and by quantitatively analyzing the correctness rate and the answer consistency of multiple responses of DeepSeek-R1 and GPT-4o API, we aim to reveal the performance differences between the two types of models in specialized, high-complexity medical exam scenarios, and provide Chinese medical educators and technology developers with a model choice for an empirical evidence basis to promote the precise and reliable application of LLMs in Chinese medical education.

## Materials and methods

### Source of questions

The test questions are extracted from the nursing (intermediate) simulation papers in the examination book designated by the Talent Exchange Service Center of the National Health and Wellness Commission of China and are in the form of objective MCQs, with a total of 400 questions divided into four units (Basic Knowledge, Relevant Professional Knowledge, Professional Knowledge, Professional Practice Ability). The basic knowledge unit examines the etiology and pathogenesis of common clinical diseases and diseases, and auxiliary examinations, of which internal medicine accounts for 35%, surgery accounts for 35%, obstetrics and gynecology accounts for 15%, and pediatrics accounts for 15%; the relevant professional knowledge examines nursing health education, hospital infection nursing, nursing management, and Chinese medicine nursing, of which nursing health education, hospital infection nursing, and nursing management each accounts for 30%, and Chinese medicine nursing accounted for 10%; professional knowledge examination of internal medicine, surgery, gynecology, pediatrics specialties of common clinical diseases, clinical manifestations, treatment points, of which internal medicine, surgical each accounted for 30%, gynecology, pediatrics accounted for 20%; professional practice ability to examine the internal medicine, surgery, obstetrics and gynecology, pediatrics comprehensive nursing content, internal medicine, surgery each accounted for 30%, gynecology, pediatrics accounted for 20%. The questions are divided into two types, A and B. Type A is a best-answer multiple-choice question; type B is a set of questions that provides a number of groups of questions, each of which shares five alternative answers, A, B, C, D, and E, listed at the front of the question, from which the answer that is most closely related to the question is chosen, and each alternative answer may be chosen once, multiple times, or not chosen.

### Model version and parameter control

We selected DeepSeek-R1 and GPT-4o (via API) for comparison based on their relevance and availability at the time of study. DeepSeek-R1 represents a new domestic large language model with increasing adoption in Chinese academic and clinical settings, while GPT-4o represents the latest widely recognized international state-of-the-art model. This pairwise comparison allowed us to examine performance differences between a cutting-edge domestic model and a global benchmark within the context of the Chinese Health Professional and Technical Examination.

Both models were configured with a temperature parameter of 0 (deterministic sampling mode) to ensure answer consistency across repeated queries [25]. Generation constraints included: ①maximum output length of 10 tokens to enforce single-character responses (A-E), ②suppression of explanatory text generation, and ③mitigation of formatting errors through restricted reasoning pathways, thereby eliminating response redundancy associated with excessive model deliberation.

### Standardized treatment

In order to ensure the standardization and comparability of the model response, this study designed a standardized prompt template for each question with the following structure: ①Mark the question stem with Clearly define the role of the model and the response requirements, the first line begins with [instruction], emphasizing the identity of the model as the “nurse in charge”, and requesting that the model “output only the letters of the options”;②[title], retaining the key clinical information and option content of the original question. “The first line begins with [instruction], emphasizing the model’s status as a “nurse practitioner in charge” and asking it to “output only the letters of the options the question stem is marked with [title], which retains the key clinical information and options in the original question. At the end of the question, it is stated in parentheses “(Answer Requirement: Output Option Letters Only)” to strengthen the output format restriction.; [2] ③ Remove the marking symbols in the original question and unify the Chinese and English punctuation to ensure the consistency of the input text; if the options in the original question contain alphabetic suffixes, they will be changed to consecutive capital letters; if the model outputs non-alphabetic characters, the first letter will be extracted automatically; if more than one letter is output, it will be regarded as an error in the format and marked as an invalid response.To enhance transparency and reproducibility, representative examples of the standardized prompt template and model outputs are provided in the S1 Fig. This approach was designed to evaluate the model’s factual knowledge based on its ability to select the correct answer without introducing confounding variables such as reasoning complexity or verbose explanations. By constraining the output to a single letter, we minimize the potential for variability in reasoning styles, focusing purely on whether the model “knows” the correct answer.

### Assessment of indicators

#### Primary outcomes.

The primary evaluation metrics comprised overall accuracy, consistency, and consistent accuracy. Overall accuracy was calculated as the percentage of correctly answered questions relative to the total administered items (Correct Responses/ Total Questions × 100%). Consistency was defined as the reproducibility of responses across repeated trials, regardless of correctness. Each question was presented to each model five times. A model’s response was considered consistent if it provided the same option for at least 4 out of 5 repetitions (≥80% threshold). This metric reflects the stability of a model’s output generation process. Consistent accuracy was defined as the percentage of questions for which the model demonstrated both consistency and correctness (i.e., stable and correct responses across repeated trials). This metric integrates reliability with validity, thereby distinguishing between merely stable errors and genuinely reliable correctness. This distinction is critical: a model with high consistency but low consistent accuracy may repeatedly generate the same incorrect answer, reflecting systematic error rather than true reliability.

#### Secondary outcomes.

Secondary metrics included stratified analyses of overall accuracy and consistent accuracy. Hierarchical comparisons were made by test unit, question type, and discipline to evaluate model performance across different domains.

### Statistical analysis

Descriptive statistics were used to summarize model performance across all evaluation metrics. Categorical variables—including accuracy, consistency, and consistent accuracy—were presented as frequencies and percentages (n, %). Consistency was defined as agreement across at least 4 of 5 repeated responses for each item (80% threshold). Consistent accuracy was calculated as the proportion of items for which responses were both consistent and correct. Chi-square tests (α = 0.05) were used to compare performance differences between DeepSeek-R1 and the GPT-4o API across examination units, question types, and clinical disciplines. Because the stratified analyses involved multiple simultaneous comparisons, Holm–Bonferroni correction was applied to adjust p-values and control the family-wise error rate. Raw p-values were used for overall accuracy comparisons and graphical visualizations, whereas adjusted p-values were reported for consistency-based analyses.Invalid responses caused by format errors (e.g., outputs not conforming to single-letter requirements) were counted and excluded from comparative statistical analyses but reported descriptively. All preprocessing was conducted in Microsoft Excel 2021, and statistical tests were performed using SPSS version 29.0. Statistical significance was interpreted using conventional thresholds (***p < 0.001, **p < 0.01, *p < 0.05).

### Ethics approval and consent to participate

This research utilized exclusively publicly accessible data from non-sensitive sources, encompassing neither human subjects, confidential health records, nor animal experimentation. As such, the research complies with the World Medical Association Declaration of Helsinki guidelines for non-interventional studies involving publicly available information, thereby exempt from institutional ethics review board approval requirements.

## Results

**Fig 1:**
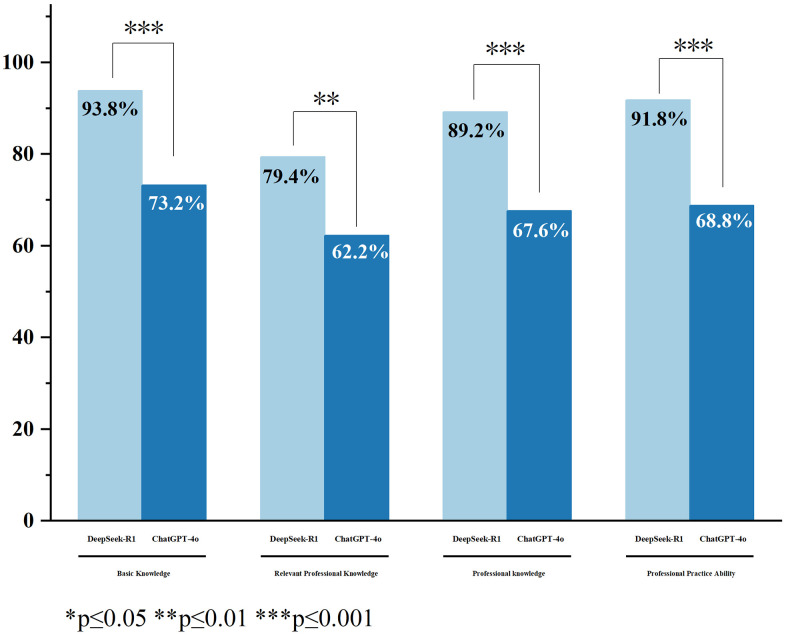
Comparison of overall accuracy of DeepSeek-R1 and GPT-4o API in different units. For overall accuracy by question type, DeepSeek-R1 achieved 89.1% accuracy on Type A questions compared with 69.1% for the GPT-4o API (P < 0.001), and 86.5% versus 64.0% on Type B questions (P = 0.001), indicating significant differences between the two models across both formats.

The results showed that DeepSeek-R1 achieved a significantly higher overall accuracy (88.5%) than the GPT-4o API (67.9%; *P* < 0.001). Fig 1 presents the overall accuracy distribution across the four examination units. In both models, accuracy was highest in the Basic Knowledge unit (93.8% for DeepSeek-R1 and 73.2% for GPT-4o API) and lowest in Relevant Professional Knowledge (79.4% and 62.2%, respectively). Using raw p-values for overall accuracy comparisons within units (as these analyses involved only descriptive accuracy metrics rather than consistency-based repeated measures), DeepSeek-R1 demonstrated significantly higher accuracy than GPT-4o API in all four units: Basic Knowledge (P < 0.001), Professional Knowledge (*P* < 0.001), Professional Practice Ability (*P* < 0.001), and Relevant Professional Knowledge (*P* = 0.008).

**Fig 2:**
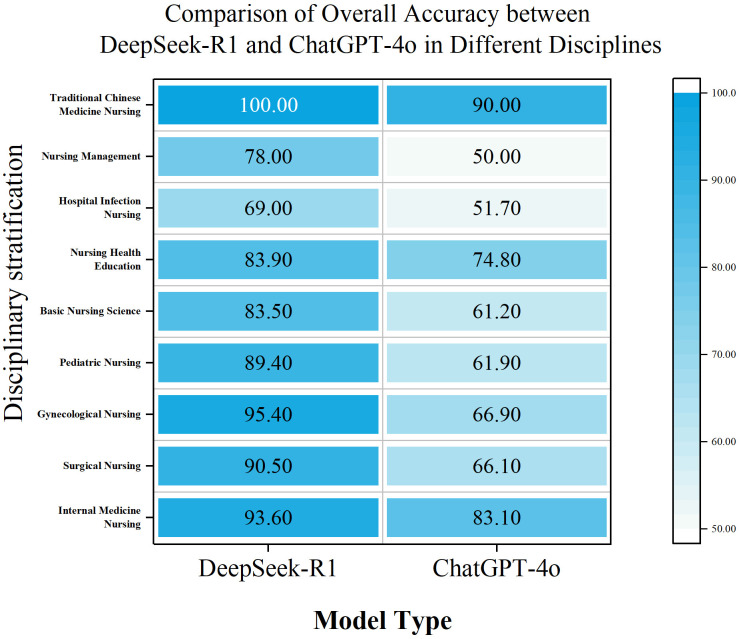
Comparison of overall accuracy between DeepSeek-R1 and GPT-4o API in different disciplines. We conducted a comparative analysis of the two models’ consistency, and the results showed that GPT-4o API was more consistent, with a consistency rate of 96.5%. The consistency rate of DeepSeek-R1 was 88.5%, and there was a significant difference between the two (*P* < 0.001). However, we also found that GPT-4o API’s consistent accuracy rate was only 66.7%, while DeepSeek-R1’s was 84.0%. This difference was also significant, *P* < 0.001.

We also compared the overall accuracy of the two models in different disciplines, and the results, as shown in Fig 2, showed that the two models differed significantly in internal medicine nursing (*n* = 83, *P* = 0.028), surgical nursing (*n* = 95, *P* < 0.001), gynecological nursing (*n* = 52, *P* < 0.001), pediatric nursing (*n* = 53, *P* = 0.002), and nursing management (*n* = 30, *P* = 0.032). In contrast, there were no significant in four disciplines: basic nursing (*n* = 17, *P* = 0.132), nursing health education (*n* = 31, *P* = 0.349), hospital infection nursing (*n* = 29, *P* = 0.180), and Chinese medicine nursing (*n* = 10, *P* = 0.305).

The results of consistent accuracy across different units, question types, and clinical disciplines are summarized in Table 1. After applying the Holm–Bonferroni correction for multiple comparisons, significant differences between DeepSeek-R1 and GPT-4o API remained in two of the four unit domains: basic knowledge (adjusted p = 0.004) and professional knowledge (adjusted p = 0.032). Professional practice ability also remained statistically significant after correction (adjusted p = 0.040), whereas relevant professional knowledge continued to show no significant difference between the models. When analyzed by question type, the difference in consistent accuracy remained highly significant for Type A questions (adjusted p < 0.001). In contrast, the difference for Type B questions did not remain statistically significant after correction (adjusted p = 0.073), although the direction of effect remained consistent with the unadjusted analysis. Across clinical disciplines, DeepSeek-R1 and GPT-4o API exhibited significant differences prior to adjustment in surgical nursing, gynecological nursing, and pediatric nursing. After applying the Holm correction, significant differences persisted in surgical nursing (adjusted p = 0.044) and gynecological nursing (adjusted p = 0.023), while the difference in pediatric nursing did not remain statistically significant (adjusted p = 0.080). No significant differences were observed in internal medicine nursing, basic nursing, nursing health education, hospital infection nursing, nursing management, or Chinese medicine nursing after adjustment.

**Table 1:** Comparison of consistent accuracy between DeepSeek-R1 and GPT-4o API in different categorizations.

Categorization	DeepSeek-R1 consistent answers, *n* (%)	GPT-4o API consistent answer, *n* (%)	Total, *n* (%)	*χ* ^*2*^	*P*-value	*Adjusted P-value*
**By different units**						
Basic Knowledge	92 (92.0)	71 (71.0)	100 (25.0)	14.624	<0.001***	0.004**
Relevant Professional Knowledge	72 (72.0)	60 (60.0)	100 (25.0)	3.209	0.073	0.514
Professional Knowledge	86 (86.0)	67 (67.0)	100 (25.0)	10.040	0.002**	0.032*
Professional Practice Ability	86 (86.0)	69 (69.0)	100 (25.0)	8.287	0.004**	0.040*
**By type of question**						
Type A questions	272 (85.0)	218 (68.1)	320 (80.0)	25.391	<0.001***	<0.001***
Type B questions	64 (80.0)	49 (61.2)	80 (20.0)	6.778	0.009**	0.073
**By different disciplines**						
Internal Medicine Nursing	76 (91.6)	68 (81.9)	83 (20.8)	3.354	0.067	0.406
Surgical Nursing	81 (85.3)	63 (66.3)	95 (23.8)	9.293	0.002**	0.044*
Gynecological Nursing	48 (92.3)	34 (65.4)	52 (13)	11.299	<0.001***	0.023*
Pediatric Nursing	45 (84.9)	32 (60.4)	53 (13.3)	8.022	0.005**	0.080
Basic Nursing	14 (82.4)	10 (58.8)	17 (4.3)	2.267	0.132	0.529
Nursing Health Education	24 (77.4)	22 (71)	31 (7.8)	0.337	0.562	1.000
Hospital Infection Nursing	18 (62.1)	15 (51.7)	29 (7.3)	0.633	0.426	1.000
Nursing Management	20 (66.7)	14 (46.7)	30 (7.5)	2.443	0.118	0.589
Chinese Medicine Nursing	10 (100.0)	9 (90.0)	10 (2.5)	0.000	1.000	1.000

Note: Chi-square tests were used to compare consistent accuracy between models for each category. P-values were adjusted for multiple comparisons using the Holm–Bonferroni method (m = 15). Significance levels: ***p < 0.001, **p < 0.01, *p < 0.05.

## Discussion

### Comparison of advantages and disadvantages and analysis of causes

The superior overall accuracy of DeepSeek-R1 suggests that it may have stronger alignment with the linguistic and conceptual structure of the Chinese nursing examination, as well as potentially more effective representation of Chinese medical text. While the specific training corpus of DeepSeek-R1 is not publicly disclosed, it is plausible that domain-relevant content or Chinese-language clinical materials contributed to its performance advantages. For Chinese nursing education and clinical knowledge updating, such characteristics may allow DeepSeek-R1 to provide comparatively more reliable factual references.In contrast, although GPT-4o API demonstrated higher response consistency, its consistently correct rate was substantially lower. This pattern indicates the presence of “stable but incorrect” responses, reflecting systematic rather than random error. Prior research has shown that systematic LLM errors can reinforce misconceptions among learners and introduce biased or unsafe content into educational materials [26]. In clinical decision-support scenarios, repeated incorrect outputs may pose risks if users rely on them without verification, particularly in high-stakes domains such as drug dosage calculation or diagnostic reasoning. DeepSeek-R1, by achieving both high consistency and high consistent accuracy, appears more reliable in this regard. These findings underscore the importance of evaluating not only model stability but also the correctness of stable responses. Accordingly, healthcare professionals should apply LLM output with critical appraisal and contextual judgment to safeguard patient safety.Differences across disciplinary domains may reflect variations in model sensitivity to specific medical knowledge structures. After adjustment for multiple comparisons, DeepSeek-R1 retained significant advantages in the basic knowledge unit as well as in surgical and gynecological nursing, suggesting greater robustness in areas characterized by standardized procedures and structured clinical reasoning. Disciplines such as pediatrics showed a trend favoring DeepSeek-R1, although statistical significance was not retained after correction, indicating that additional data may be needed to confirm domain-specific differences. Other domains, including basic nursing, nursing health education, hospital infection nursing, and Chinese medicine nursing, showed no significant differences between models, which may relate to their broader conceptual knowledge bases or, in the case of Chinese medicine nursing, the presence of culturally specific theoretical content that both models may represent only partially [27]. he performance differences observed between DeepSeek-R1 and GPT-4o may also relate to differences in model architecture and semantic processing, as prior studies suggest that LLMs vary in their handling of complex logical dependencies and domain-rich language [28]. Future research could further investigate which architectural features or training strategies enhance model performance on medical text, including analyses of error patterns, linguistic sensitivity, and domain-specific reasoning pathways.

### Differences in the suitability of disciplines and question types

After multiple-comparison adjustment, DeepSeek-R1 continued to show stronger performance than GPT-4o in the clinical disciplines of surgical nursing and gynecological nursing, suggesting that its training may better capture procedural knowledge, structured clinical pathways, and guideline-based decision-making. These domains typically involve clear diagnostic frameworks and standardized nursing interventions, which may align more closely with DeepSeek-R1’s model characteristics. This pattern provides practical implications for medical education: DeepSeek-R1 may serve as a more reliable auxiliary tool when teaching clinically structured content that requires procedural accuracy.In contrast, disciplines such as basic nursing, internal medicine nursing, nursing management, hospital infection nursing, and Chinese medicine nursing did not show significant differences between the two models after correction. The lack of performance gap may stem from the nature of these domains, which contain a higher proportion of generalizable principles or culturally specific knowledge (e.g., in Chinese medicine nursing), where both models may have limited or similar depth of domain representation. For areas grounded in traditional theory, existing LLMs may require more targeted training data or domain-specific adaptation to achieve meaningful improvements [29].Regarding question types, DeepSeek-R1’s advantage remained statistically significant for Type A questions (standardized, single-scenario items), indicating stronger stability in structured reasoning and factual recall. Although DeepSeek-R1 also outperformed GPT-4o in Type B questions (complex, multi-clue scenarios), the difference did not remain significant after Holm correction. Nevertheless, the directional trend still suggests that DeepSeek-R1 may hold greater potential in handling integrated clinical information, an ability that is essential in real-world nursing where dynamic and multifaceted clinical cues must be synthesized for decision-making [30]. GPT-4o’s comparatively lower performance in standardized items indicates room for optimization in addressing straightforward factual content, and the contrast between the two models reflects fundamental differences in their problem-solving strategies. These differences provide valuable insights for model refinement and for selecting appropriate LLMs for specific educational or clinical tasks.

### Clinical application risks and coping strategies

The risk posed by GPT-4o API’s low consistent correctness, despite its high response agreement, warrants close attention. The phenomenon of “stable but wrong” outputs carries different implications across settings. In educational contexts, repeated exposure to incorrect answers may reinforce learner misconceptions and hinder the development of accurate clinical knowledge structures. In clinical environments, however, the potential consequences are more serious. For domains such as hospital infection prevention and control, recurrent erroneous associations could increase the risk of unsafe practices if users adopt model outputs uncritically, thereby posing threats to patient and staff safety and potentially contributing to malpractice events [31]. These findings illustrate that high consistency does not equate to reliability, and systematic errors may be more hazardous than random inaccuracies.

Given these risks, GPT-4o API should ideally be paired with a human auditing mechanism. Yet manual auditing is time-intensive, resource-demanding, and subject to variability in evaluators’ professional expertise [32]. To improve efficiency, the development of intelligent auditing tools—such as systems incorporating rule engines or knowledge-graph–based verification—may offer a scalable approach for automated error detection [33]. The proposed “Dual Model Cross-Validation” strategy represents another potential safeguard by leveraging complementary strengths of different LLMs. However, in addition to technical challenges such as managing data interchange and resolving conflicting outputs, practical considerations must be addressed. These include increased computational burden, integration into existing clinical or educational workflows, and the continued need for human oversight when models disagree. One feasible direction may be to apply dual-model validation selectively in high-stakes scenarios—such as medication safety, infection control, or ethically sensitive decision-making—while allowing single-model outputs to support lower-stakes learning tasks [34]. Future research should evaluate the cost-effectiveness, usability, and real-world safety implications of multi-model validation frameworks in authentic teaching and clinical environments. Systematic assessments using real clinical workflows will be essential to determine whether such strategies can meaningfully mitigate the risks associated with systematic LLM errors.

### Research limitations and future research directions

This study has several limitations that should be acknowledged. First, only multiple-choice questions (MCQs) were used, representing a single question type that mainly assesses recognition-based factual knowledge. This narrow scope does not capture higher-order competencies such as open-ended clinical reasoning, care plan development, or ethical decision-making, which are central to nursing practice. Consequently, the ecological validity of our findings is limited: real-world nursing work often requires context-sensitive judgment, nuanced patient communication, and ethical deliberation, all of which extend beyond the ability to select a correct option.

Future studies should therefore expand the scope of evaluation by incorporating open-ended clinical problems and ethical decision-making scenarios. For open-ended items, performance could be assessed in terms of completeness, accuracy, and logical coherence, while ethical decision-making could be evaluated against established professional guidelines and expert consensus. In addition, extending the assessment to other medical specialty examinations (e.g., clinical medicine, pharmacy) would help determine whether model performance varies across domains and provide more targeted insights for medical education and clinical practice.Second, this study compared only two models (DeepSeek-R1 and GPT-4o). The absence of additional baselines such as GPT-3.5, Claude, or Gemini limits the generalizability of our conclusions. Including a wider range of large language models in future research would provide a more comprehensive benchmark and allow cross-family comparisons. Accordingly, our results should be interpreted as a comparative case study between two representative systems rather than an exhaustive evaluation of all available LLMs.Third, the data source of test items also imposes constraints. Since the official national nursing licensure examination items are not publicly released, we could not directly evaluate model performance on the true exam bank. Instead, our study relied on the officially designated practice questions, which are constructed according to the national exam syllabus and serve as the standard preparation resource for candidates. Although these items reflect the content scope and expected competency level of the official examination, they do not undergo the same psychometric validation process (e.g., calibration of item difficulty, discrimination, fairness, exposure control). As a result, the generalizability of our findings to the actual exam should be interpreted with caution. Future work would benefit from collaboration with examination authorities to access psychometrically validated items, thereby providing stronger evidence of model performance under authentic testing conditions.

## Conclusion

This study demonstrated that DeepSeek-R1 outperformed the GPT-4o API in overall accuracy, consistency, and consistent accuracy on the Chinese Health Professional and Technical Examination (Intermediate Nursing). DeepSeek-R1 showed particular strengths in basic knowledge and selected clinical disciplines, whereas the GPT-4o API exhibited high consistency but a substantially lower rate of consistently correct responses, indicating a tendency toward systematic rather than random error. These findings highlight the importance of evaluating both accuracy and response stability when considering large language models for use in medical education. Although the results suggest that DeepSeek-R1 may offer more reliable support for fact-based or structured nursing content, model outputs should be used cautiously in educational or clinical settings, particularly in high-stakes domains. Further research is needed to assess model behavior using open-ended clinical reasoning tasks, real-world examination data, and authentic clinical workflows, and to determine the feasibility and safety of approaches such as multi-model validation or automated audit mechanisms. Continued empirical evaluation will be essential to ensure that LLMs can be integrated responsibly and effectively into medical education and clinical training.

## Supporting information

S1 Fig Examples of standardized prompts and model outputs. (PNG)

S1 Appendix SPSS statistical output for model performance comparisons. (DOCX)

S2 Appendix Dataset used for figures and statistical analyses. (XLSX)

## Abbreviations

AI: artificial intelligence
LLMs: large language models
MCQs: multiple-choice questions
