# Supplementary figures and images for: Performance of DeepSeek and ChatGPT on the Chinese Health Professional and Technical Examination: A comparative study
Source: PLoS One. 2026 Jan 22;21(1):e0338328. doi: 10.1371/journal.pone.0338328 (PMC12826474; doi:10.1371/journal.pone.0338328)

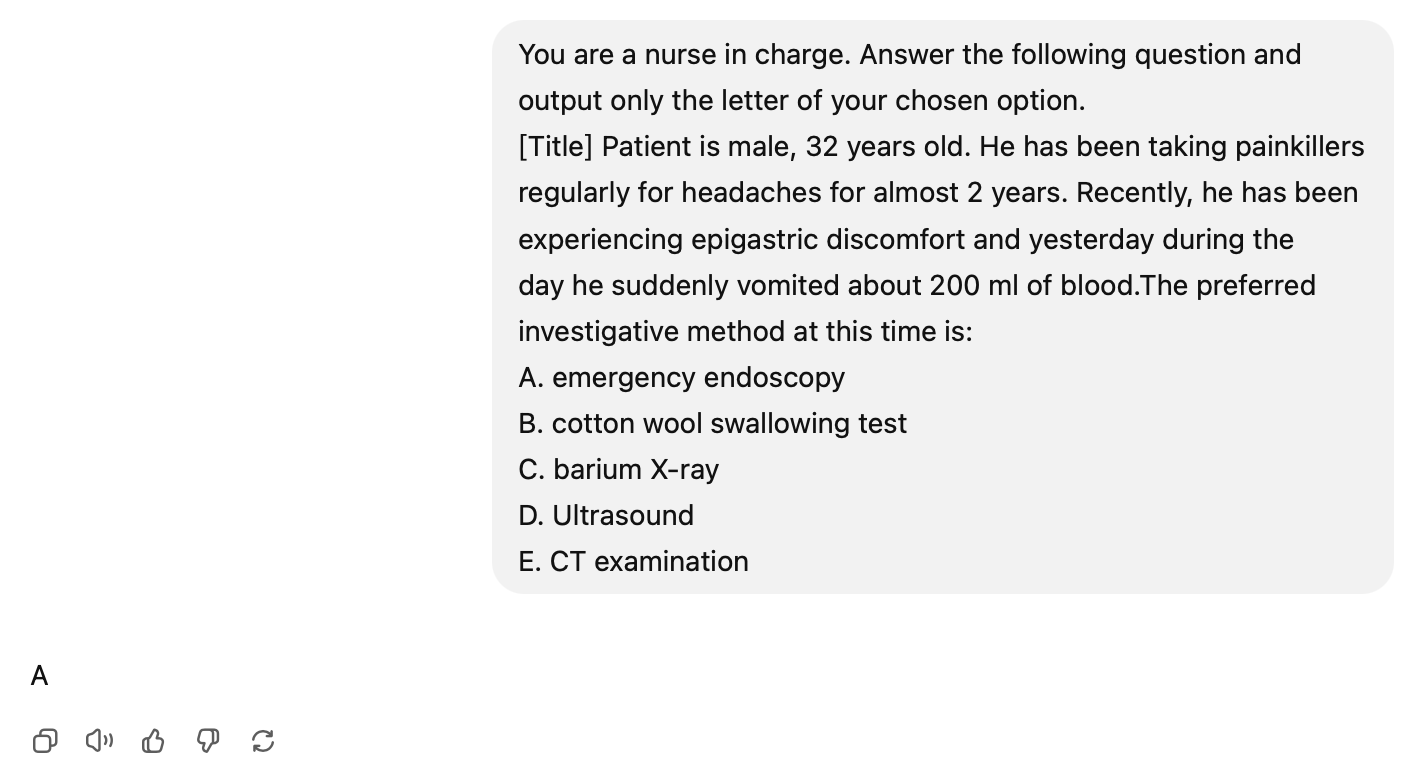

Supplement: S1 Fig — (PNG) [file pone.0338328.s001.png]
