# Supplementary material for: Performance of DeepSeek and ChatGPT on the Chinese Health Professional and Technical Examination: A comparative study
Source: PLoS One. 2026 Jan 22;21(1):e0338328. doi: 10.1371/journal.pone.0338328 (PMC12826474; doi:10.1371/journal.pone.0338328)
Supplement: S1 Appendix — (DOCX) [file pone.0338328.s002.docx]

| **Case Summary** | | | | | | | |
| --- | --- | --- | --- | --- | --- | --- | --- |
| Category | | individual cases | | | | | |
|  |  | valid | | hiatus | | amount to | |
|  |  | N | percentage | N | percentage | N | percentage |
| Basic Knowledge | Model * Response | 200 | 100.0% | 0 | 0.0% | 200 | 100.0% |
| Basic Nursing | Model * Response | 34 | 100.0% | 0 | 0.0% | 34 | 100.0% |
| Chinese Medicine Nursing | Model * Response | 20 | 100.0% | 0 | 0.0% | 20 | 100.0% |
| Gynecological Nursing | Model * Response | 104 | 100.0% | 0 | 0.0% | 104 | 100.0% |
| Hospital Infection Nursing | Model * Response | 58 | 100.0% | 0 | 0.0% | 58 | 100.0% |
| Internal Medicine Nursing | Model * Response | 166 | 100.0% | 0 | 0.0% | 166 | 100.0% |
| Nursing Health Education | Model * Response | 62 | 100.0% | 0 | 0.0% | 62 | 100.0% |
| Nursing Management | Model * Response | 60 | 100.0% | 0 | 0.0% | 60 | 100.0% |
| Pediatric Nursing | Model * Response | 106 | 100.0% | 0 | 0.0% | 106 | 100.0% |
| Professional Knowledge | Model * Response | 200 | 100.0% | 0 | 0.0% | 200 | 100.0% |
| Professional Practice Ability | Model * Response | 200 | 100.0% | 0 | 0.0% | 200 | 100.0% |
| Relevant Professional Knowledge | Model * Response | 200 | 100.0% | 0 | 0.0% | 200 | 100.0% |
| Surgical Nursing | Model * Response | 190 | 100.0% | 0 | 0.0% | 190 | 100.0% |
| Type A Questions | Model * Response | 640 | 100.0% | 0 | 0.0% | 640 | 100.0% |
| Type B Questions | Model * Response | 160 | 100.0% | 0 | 0.0% | 160 | 100.0% |

| **Model * Response Cross Table** | | | | | | |
| --- | --- | --- | --- | --- | --- | --- |
| Category | | | | Response | | amount to |
|  |  |  |  | Correct | Incorrect |  |
| Basic Knowledge | Model | DeepSeek-R1 | count | 92 | 8 | 100 |
|  |  |  | Expected count | 81.5 | 18.5 | 100.0 |
|  |  |  | Percentage of Model | 92.0% | 8.0% | 100.0% |
|  |  |  | Percentage of responses | 56.4% | 21.6% | 50.0% |
|  |  | GPT-4o API | count | 71 | 29 | 100 |
|  |  |  | Expected count | 81.5 | 18.5 | 100.0 |
|  |  |  | Percentage of Model | 71.0% | 29.0% | 100.0% |
|  |  |  | Percentage of Response | 43.6% | 78.4% | 50.0% |
|  | amount to | | count | 163 | 37 | 200 |
|  |  |  | Expected count | 163.0 | 37.0 | 200.0 |
|  |  |  | Percentage of Model | 81.5% | 18.5% | 100.0% |
|  |  |  | Percentage of responses | 100.0% | 100.0% | 100.0% |
| Basic Nursing | Model | DeepSeek-R1 | count | 14 | 3 | 17 |
|  |  |  | Expected count | 12.0 | 5.0 | 17.0 |
|  |  |  | Percentage of Model | 82.4% | 17.6% | 100.0% |
|  |  |  | Percentage of responses | 58.3% | 30.0% | 50.0% |
|  |  | GPT-4o API | count | 10 | 7 | 17 |
|  |  |  | Expected count | 12.0 | 5.0 | 17.0 |
|  |  |  | Percentage of Model | 58.8% | 41.2% | 100.0% |
|  |  |  | Percentage of responses | 41.7% | 70.0% | 50.0% |
|  | amount to | | count | 24 | 10 | 34 |
|  |  |  | Expected count | 24.0 | 10.0 | 34.0 |
|  |  |  | Percentage of Model | 70.6% | 29.4% | 100.0% |
|  |  |  | Percentage of responses | 100.0% | 100.0% | 100.0% |
| Chinese Medicine Nursing | Model | DeepSeek-R1 | count | 10 | 0 | 10 |
|  |  |  | Expected count | 9.5 | .5 | 10.0 |
|  |  |  | Percentage of Model | 100.0% | 0.0% | 100.0% |
|  |  |  | Percentage of responses | 52.6% | 0.0% | 50.0% |
|  |  | GPT-4o API | count | 9 | 1 | 10 |
|  |  |  | Expected count | 9.5 | .5 | 10.0 |
|  |  |  | Percentage of Model | 90.0% | 10.0% | 100.0% |
|  |  |  | Percentage of responses | 47.4% | 100.0% | 50.0% |
|  | amount to | | count | 19 | 1 | 20 |
|  |  |  | Expected count | 19.0 | 1.0 | 20.0 |
|  |  |  | Percentage of Model | 95.0% | 5.0% | 100.0% |
|  |  |  | Percentage of responses | 100.0% | 100.0% | 100.0% |
| Gynecological Nursing | Model | DeepSeek-R1 | count | 48 | 4 | 52 |
|  |  |  | Expected count | 41.0 | 11.0 | 52.0 |
|  |  |  | Percentage of Model | 92.3% | 7.7% | 100.0% |
|  |  |  | Percentage of responses | 58.5% | 18.2% | 50.0% |
|  |  | GPT-4o API | count | 34 | 18 | 52 |
|  |  |  | Expected count | 41.0 | 11.0 | 52.0 |
|  |  |  | Percentage of Model | 65.4% | 34.6% | 100.0% |
|  |  |  | Percentage of responses | 41.5% | 81.8% | 50.0% |
|  | amount to | | count | 82 | 22 | 104 |
|  |  |  | Expected count | 82.0 | 22.0 | 104.0 |
|  |  |  | Percentage of Model | 78.8% | 21.2% | 100.0% |
|  |  |  | Percentage of responses | 100.0% | 100.0% | 100.0% |
| Hospital Infection Nursing | Model | DeepSeek-R1 | count | 18 | 11 | 29 |
|  |  |  | Expected count | 16.5 | 12.5 | 29.0 |
|  |  |  | Percentage of Model | 62.1% | 37.9% | 100.0% |
|  |  |  | Percentage of responses | 54.5% | 44.0% | 50.0% |
|  |  | GPT-4o API | count | 15 | 14 | 29 |
|  |  |  | Expected count | 16.5 | 12.5 | 29.0 |
|  |  |  | Percentage of Model | 51.7% | 48.3% | 100.0% |
|  |  |  | Percentage of responses | 45.5% | 56.0% | 50.0% |
|  | amount to | | count | 33 | 25 | 58 |
|  |  |  | Expected count | 33.0 | 25.0 | 58.0 |
|  |  |  | Percentage of Model | 56.9% | 43.1% | 100.0% |
|  |  |  | Percentage of responses | 100.0% | 100.0% | 100.0% |
| Internal Medicine Nursing | Model | DeepSeek-R1 | count | 76 | 7 | 83 |
|  |  |  | Expected count | 72.0 | 11.0 | 83.0 |
|  |  |  | Percentage of Model | 91.6% | 8.4% | 100.0% |
|  |  |  | Percentage of responses | 52.8% | 31.8% | 50.0% |
|  |  | GPT-4o API | count | 68 | 15 | 83 |
|  |  |  | Expected count | 72.0 | 11.0 | 83.0 |
|  |  |  | Percentage of Model | 81.9% | 18.1% | 100.0% |
|  |  |  | Percentage of responses | 47.2% | 68.2% | 50.0% |
|  | amount to | | count | 144 | 22 | 166 |
|  |  |  | Expected count | 144.0 | 22.0 | 166.0 |
|  |  |  | Percentage of Model | 86.7% | 13.3% | 100.0% |
|  |  |  | Percentage of responses | 100.0% | 100.0% | 100.0% |
| Nursing Health Education | Model | DeepSeek-R1 | count | 24 | 7 | 31 |
|  |  |  | Expected count | 23.0 | 8.0 | 31.0 |
|  |  |  | Percentage of Model | 77.4% | 22.6% | 100.0% |
|  |  |  | Percentage of responses | 52.2% | 43.8% | 50.0% |
|  |  | GPT-4o API | count | 22 | 9 | 31 |
|  |  |  | Expected count | 23.0 | 8.0 | 31.0 |
|  |  |  | Percentage of Model | 71.0% | 29.0% | 100.0% |
|  |  |  | Percentage of responses | 47.8% | 56.3% | 50.0% |
|  | amount to | | count | 46 | 16 | 62 |
|  |  |  | Expected count | 46.0 | 16.0 | 62.0 |
|  |  |  | Percentage of Model | 74.2% | 25.8% | 100.0% |
|  |  |  | Percentage of responses | 100.0% | 100.0% | 100.0% |
| Nursing Management | Model | DeepSeek-R1 | count | 20 | 10 | 30 |
|  |  |  | Expected count | 17.0 | 13.0 | 30.0 |
|  |  |  | Percentage of Model | 66.7% | 33.3% | 100.0% |
|  |  |  | Percentage of responses | 58.8% | 38.5% | 50.0% |
|  |  | GPT-4o API | count | 14 | 16 | 30 |
|  |  |  | Expected count | 17.0 | 13.0 | 30.0 |
|  |  |  | Percentage of Model | 46.7% | 53.3% | 100.0% |
|  |  |  | Percentage of Response | 41.2% | 61.5% | 50.0% |
|  | amount to | | count | 34 | 26 | 60 |
|  |  |  | Expected count | 34.0 | 26.0 | 60.0 |
|  |  |  | Percentage of Model | 56.7% | 43.3% | 100.0% |
|  |  |  | Percentage of responses | 100.0% | 100.0% | 100.0% |
| Pediatric Nursing | Model | DeepSeek-R1 | count | 45 | 8 | 53 |
|  |  |  | Expected count | 38.5 | 14.5 | 53.0 |
|  |  |  | Percentage of Model | 84.9% | 15.1% | 100.0% |
|  |  |  | Percentage of responses | 58.4% | 27.6% | 50.0% |
|  |  | GPT-4o API | count | 32 | 21 | 53 |
|  |  |  | Expected count | 38.5 | 14.5 | 53.0 |
|  |  |  | Percentage of Model | 60.4% | 39.6% | 100.0% |
|  |  |  | Percentage of responses | 41.6% | 72.4% | 50.0% |
|  | amount to | | count | 77 | 29 | 106 |
|  |  |  | Expected count | 77.0 | 29.0 | 106.0 |
|  |  |  | Percentage of Model | 72.6% | 27.4% | 100.0% |
|  |  |  | Percentage of Response | 100.0% | 100.0% | 100.0% |
| Professional Knowledge | Model | DeepSeek-R1 | count | 86 | 14 | 100 |
|  |  |  | Expected count | 76.5 | 23.5 | 100.0 |
|  |  |  | Percentage of Model | 86.0% | 14.0% | 100.0% |
|  |  |  | Percentage of responses | 56.2% | 29.8% | 50.0% |
|  |  | GPT-4o API | count | 67 | 33 | 100 |
|  |  |  | Expected count | 76.5 | 23.5 | 100.0 |
|  |  |  | Percentage of Model | 67.0% | 33.0% | 100.0% |
|  |  |  | Percentage of responses | 43.8% | 70.2% | 50.0% |
|  | amount to | | count | 153 | 47 | 200 |
|  |  |  | Expected count | 153.0 | 47.0 | 200.0 |
|  |  |  | Percentage of Model | 76.5% | 23.5% | 100.0% |
|  |  |  | Percentage of responses | 100.0% | 100.0% | 100.0% |
| Professional Practice Ability | Model | DeepSeek-R1 | count | 86 | 14 | 100 |
|  |  |  | Expected count | 77.5 | 22.5 | 100.0 |
|  |  |  | Percentage of Model | 86.0% | 14.0% | 100.0% |
|  |  |  | Percentage of responses | 55.5% | 31.1% | 50.0% |
|  |  | GPT-4o API | count | 69 | 31 | 100 |
|  |  |  | Expected count | 77.5 | 22.5 | 100.0 |
|  |  |  | Percentage of Model | 69.0% | 31.0% | 100.0% |
|  |  |  | Percentage of responses | 44.5% | 68.9% | 50.0% |
|  | amount to | | count | 155 | 45 | 200 |
|  |  |  | Expected count | 155.0 | 45.0 | 200.0 |
|  |  |  | Percentage of Model | 77.5% | 22.5% | 100.0% |
|  |  |  | Percentage of responses | 100.0% | 100.0% | 100.0% |
| Relevant Professional Knowledge | Model | DeepSeek-R1 | count | 72 | 28 | 100 |
|  |  |  | Expected count | 66.0 | 34.0 | 100.0 |
|  |  |  | Percentage of Model | 72.0% | 28.0% | 100.0% |
|  |  |  | Percentage of responses | 54.5% | 41.2% | 50.0% |
|  |  | GPT-4o API | count | 60 | 40 | 100 |
|  |  |  | Expected count | 66.0 | 34.0 | 100.0 |
|  |  |  | Percentage of Model | 60.0% | 40.0% | 100.0% |
|  |  |  | Percentage of responses | 45.5% | 58.8% | 50.0% |
|  | amount to | | count | 132 | 68 | 200 |
|  |  |  | Expected count | 132.0 | 68.0 | 200.0 |
|  |  |  | Percentage of Model | 66.0% | 34.0% | 100.0% |
|  |  |  | Percentage of responses | 100.0% | 100.0% | 100.0% |
| Surgical Nursing | Model | DeepSeek-R1 | count | 81 | 14 | 95 |
|  |  |  | Expected count | 72.0 | 23.0 | 95.0 |
|  |  |  | Percentage of Model | 85.3% | 14.7% | 100.0% |
|  |  |  | Percentage of responses | 56.3% | 30.4% | 50.0% |
|  |  | GPT-4o API | count | 63 | 32 | 95 |
|  |  |  | Expected count | 72.0 | 23.0 | 95.0 |
|  |  |  | Percentage of Model | 66.3% | 33.7% | 100.0% |
|  |  |  | Percentage of responses | 43.8% | 69.6% | 50.0% |
|  | amount to | | count | 144 | 46 | 190 |
|  |  |  | Expected count | 144.0 | 46.0 | 190.0 |
|  |  |  | Percentage of Model | 75.8% | 24.2% | 100.0% |
|  |  |  | Percentage of responses | 100.0% | 100.0% | 100.0% |
| Type A Questions | Model | DeepSeek-R1 | count | 272 | 48 | 320 |
|  |  |  | Expected count | 245.0 | 75.0 | 320.0 |
|  |  |  | Percentage of Model | 85.0% | 15.0% | 100.0% |
|  |  |  | Percentage of responses | 55.5% | 32.0% | 50.0% |
|  |  | GPT-4o API | count | 218 | 102 | 320 |
|  |  |  | Expected count | 245.0 | 75.0 | 320.0 |
|  |  |  | Percentage of Model | 68.1% | 31.9% | 100.0% |
|  |  |  | Percentage of responses | 44.5% | 68.0% | 50.0% |
|  | amount to | | count | 490 | 150 | 640 |
|  |  |  | Expected count | 490.0 | 150.0 | 640.0 |
|  |  |  | Percentage of Model | 76.6% | 23.4% | 100.0% |
|  |  |  | Percentage of responses | 100.0% | 100.0% | 100.0% |
| Type B Questions | Model | DeepSeek-R1 | count | 64 | 16 | 80 |
|  |  |  | Expected count | 56.5 | 23.5 | 80.0 |
|  |  |  | Percentage of Model | 80.0% | 20.0% | 100.0% |
|  |  |  | Percentage of responses | 56.6% | 34.0% | 50.0% |
|  |  | GPT-4o API | count | 49 | 31 | 80 |
|  |  |  | Expected count | 56.5 | 23.5 | 80.0 |
|  |  |  | Percentage of Model | 61.3% | 38.8% | 100.0% |
|  |  |  | Percentage of responses | 43.4% | 66.0% | 50.0% |
|  | amount to | | count | 113 | 47 | 160 |
|  |  |  | Expected count | 113.0 | 47.0 | 160.0 |
|  |  |  | Percentage of Model | 70.6% | 29.4% | 100.0% |
|  |  |  | Percentage of responses | 100.0% | 100.0% | 100.0% |

| **chi-square test** | | | | | | |
| --- | --- | --- | --- | --- | --- | --- |
| Category | | price | free degree | Stepwise significance (two-sided) | Precise significance (two-sided) | Significance (single side) |
| Basic Knowledge | Pearson's chi-square | ^14.624a^ | 1 | <.001 |  |  |
|  | ^Continuous Correction b^ | 13.265 | 1 | <.001 |  |  |
|  | likelihood ratio | 15.372 | 1 | <.001 |  |  |
|  | Fisher's exact test |  |  |  | <.001 | <.001 |
|  | Number of valid cases | 200 |  |  |  |  |
| Basic Nursing | Pearson's chi-square | ^2.267c^ | 1 | .132 |  |  |
|  | ^Continuous correction b^ | 1.275 | 1 | .259 |  |  |
|  | likelihood ratio | 2.315 | 1 | .128 |  |  |
|  | Fisher's exact test |  |  |  | .259 | .129 |
|  | Number of valid cases | 34 |  |  |  |  |
| Chinese Medicine Nursing | Pearson's chi-square | ^1.053d^ | 1 | .305 |  |  |
|  | ^Continuous correction b^ | .000 | 1 | 1.000 |  |  |
|  | likelihood ratio | 1.439 | 1 | .230 |  |  |
|  | Fisher's exact test |  |  |  | 1.000 | .500 |
|  | Number of valid cases | 20 |  |  |  |  |
| Gynecological Nursing | Pearson's chi-square | ^11.299e^ | 1 | <.001 |  |  |
|  | ^Continuous correction b^ | 9.743 | 1 | .002 |  |  |
|  | likelihood ratio | 12.038 | 1 | <.001 |  |  |
|  | Fisher's exact test |  |  |  | .001 | <.001 |
|  | Number of valid cases | 104 |  |  |  |  |
| Hospital Infection Nursing | Pearson's chi-square | ^.633f^ | 1 | .426 |  |  |
|  | ^Continuous correction b^ | .281 | 1 | .596 |  |  |
|  | likelihood ratio | .634 | 1 | .426 |  |  |
|  | Fisher's exact test |  |  |  | .596 | .298 |
|  | Number of valid cases | 58 |  |  |  |  |
| Internal Medicine Nursing | Pearson's chi-square | ^3.354e^ | 1 | .067 |  |  |
|  | ^Continuous Correction b^ | 2.568 | 1 | .109 |  |  |
|  | likelihood ratio | 3.422 | 1 | .064 |  |  |
|  | Fisher's exact test |  |  |  | .108 | .054 |
|  | Number of valid cases | 166 |  |  |  |  |
| Nursing Health Education | Pearson's chi-square | ^.337g^ | 1 | .562 |  |  |
|  | ^Continuous correction b^ | .084 | 1 | .772 |  |  |
|  | likelihood ratio | .338 | 1 | .561 |  |  |
|  | Fisher's exact test |  |  |  | .772 | .386 |
|  | Number of valid cases | 62 |  |  |  |  |
| Nursing Management | Pearson's chi-square | ^2.443h^ | 1 | .118 |  |  |
|  | ^Continuous correction b^ | 1.697 | 1 | .193 |  |  |
|  | likelihood ratio | 2.462 | 1 | .117 |  |  |
|  | Fisher's exact test |  |  |  | .192 | .096 |
|  | Number of valid cases | 60 |  |  |  |  |
| Pediatric Nursing | Pearson's chi-square | ^8.022i^ | 1 | .005 |  |  |
|  | ^Continuous Correction b^ | 6.836 | 1 | .009 |  |  |
|  | likelihood ratio | 8.246 | 1 | .004 |  |  |
|  | Fisher's exact test |  |  |  | .008 | .004 |
|  | Number of valid cases | 106 |  |  |  |  |
| Professional Knowledge | Pearson's chi-square | ^10.040j^ | 1 | .002 |  |  |
|  | ^Continuous correction b^ | 9.011 | 1 | .003 |  |  |
|  | likelihood ratio | 10.271 | 1 | .001 |  |  |
|  | Fisher's exact test |  |  |  | .002 | .001 |
|  | Number of valid cases | 200 |  |  |  |  |
| Professional Practice Ability | Pearson's chi-square | ^8.287k^ | 1 | .004 |  |  |
|  | ^Continuous correction b^ | 7.341 | 1 | .007 |  |  |
|  | likelihood ratio | 8.453 | 1 | .004 |  |  |
|  | Fisher's exact test |  |  |  | .006 | .003 |
|  | Number of valid cases | 200 |  |  |  |  |
| Relevant Professional Knowledge | Pearson's chi-square | ^3.209l^ | 1 | .073 |  |  |
|  | ^Continuous correction b^ | 2.696 | 1 | .101 |  |  |
|  | likelihood ratio | 3.221 | 1 | .073 |  |  |
|  | Fisher's exact test |  |  |  | .100 | .050 |
|  | Number of valid cases | 200 |  |  |  |  |
| Surgical Nursing | Pearson's chi-square | ^9.293m^ | 1 | .002 |  |  |
|  | ^Continuous Correction b^ | 8.290 | 1 | .004 |  |  |
|  | likelihood ratio | 9.491 | 1 | .002 |  |  |
|  | Fisher's exact test |  |  |  | .004 | .002 |
|  | Number of valid cases | 190 |  |  |  |  |
| Type A Questions | Pearson's chi-square | ^25.391n^ | 1 | <.001 |  |  |
|  | ^Continuous correction b^ | 24.459 | 1 | <.001 |  |  |
|  | likelihood ratio | 25.846 | 1 | <.001 |  |  |
|  | Fisher's exact test |  |  |  | <.001 | <.001 |
|  | Number of valid cases | 640 |  |  |  |  |
| Type B Questions | Pearson's chi-square | ^6.778j^ | 1 | .009 |  |  |
|  | ^Continuous correction b^ | 5.905 | 1 | .015 |  |  |
|  | likelihood ratio | 6.869 | 1 | .009 |  |  |
|  | Fisher's exact test |  |  |  | .015 | .007 |
|  | Number of valid cases | 160 |  |  |  |  |
